# Correction: Song et al. A Hydrodistillate of *Gynostemma pentaphyllum* and Damulin B Prevent Cisplatin-Induced Nephrotoxicity In Vitro and In Vivo via Regulation of AMPKα1 Transcription. *Nutrients* 2022, *14*, 4997

**DOI:** 10.3390/nu18010127

**Published:** 2025-12-31

**Authors:** Minhyeok Song, Minseok Kim, Dang Hieu Hoang, Lochana Mangesh Kovale, Jihyun Lee, Youngjoo Kim, Changhyun Lee, Jongki Hong, Sungchul Park, Wonchae Choe, Insug Kang, Sung Soo Kim, Joohun Ha

**Affiliations:** 1 Department of Biochemistry and Molecular Biology, Graduate School, College of Medicine, Kyung Hee University, Seoul 02447, Republic of Korea; thdalsgur77@naver.com (M.S.); mskim9262@naver.com (M.K.); hoang.dang.hieu.ls@gmail.com (D.H.H.); kovlelochana@gmail.com (L.M.K.); wchoe@khu.ac.kr (W.C.); iskang@khu.ac.kr (I.K.); sgskim@khu.ac.kr (S.S.K.); 2 Easy Hydrogen Corporation, Jeju City 63196, Republic of Korea; easyhydrogen@gmail.com; 3 Department of Urology, College of Medicine, Jeju National University, Jeju City 63243, Republic of Korea; kurology@jejunu.ac.kr; 4 Chunjieh Cooperation, Jeju City 63359, Republic of Korea; 7771rkstlr@hanmail.net; 5 College of Pharmacy, Kyung Hee University, Seoul 02447, Republic of Korea; jhong@khu.ac.kr (J.H.); yrs01004@naver.com (S.P.)

In the original publication [1], there was a mistake in Figure 4. The authors recognized an inadvertent error in Figure 4E (panels 4 and 5). During the process of data preparation and image assembly, part of the image may have been unintentionally duplicated between the two panels. This error likely occurred during the selection of representative images from a large number of experimental results. The corrected version of Figure 4E, panels 4 and 5, has been updated and appears below. The authors state that the scientific conclusions are unaffected. This correction was approved by the Academic Editor. The original publication has also been updated.

## Figures and Tables

**Figure 4:**
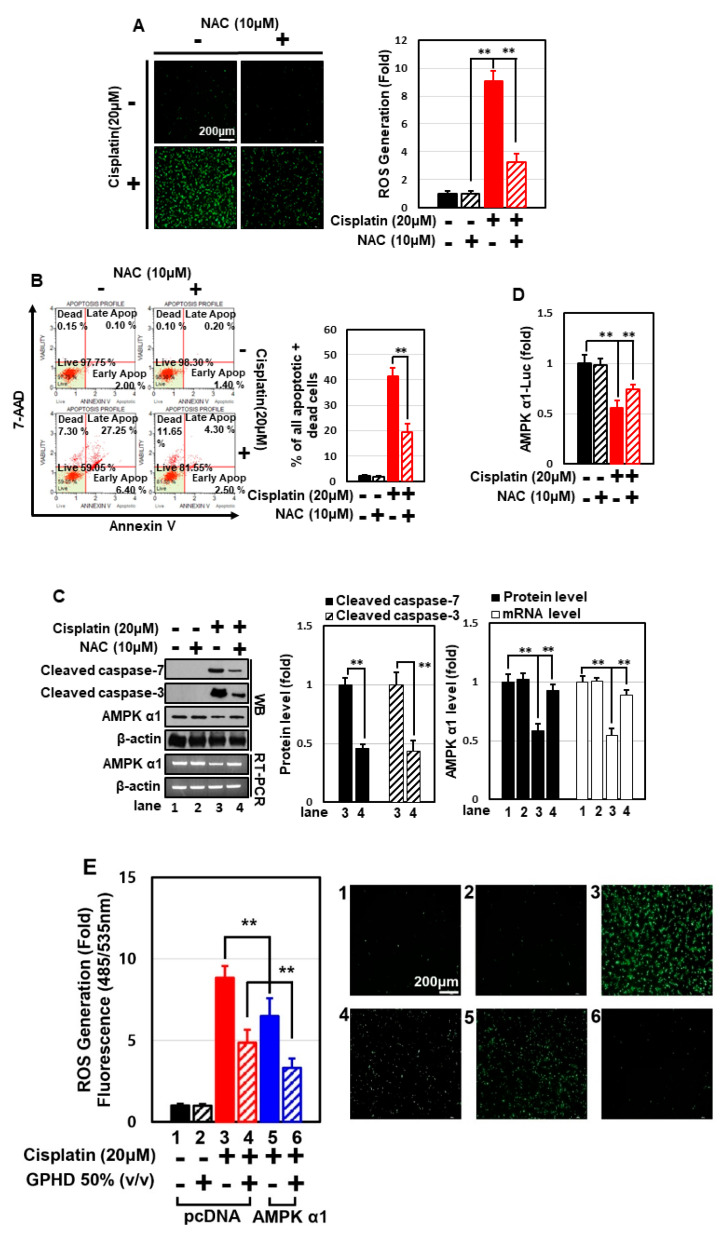

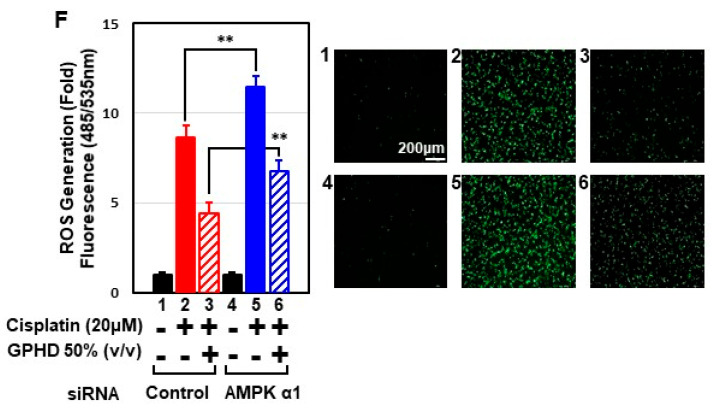
GPHD suppresses cisplatin-generated ROS and ROS-induced apoptosis via AMPKα1, a cellular anti-oxidant factor. HEK293 cells were treated with cisplatin (20 μM) and/or NAC (10 μM) for 24 h. Thereafter, intracellular ROS levels were measured (**A**), and FACS analysis of 7-AAD and annexin V double-positive cells (**B**), Western blot analysis (**C**), and luciferase reporter assays for AMPKα1 promoter activity (**D**), were performed. HEK293 cells were transfected with an expression vector for HA-tagged AMPKα1 (**E**) or siRNA for AMPKα1 (**F**), then treated with cisplatin and/or GPHD for 24 h, after which cellular ROS levels were measured. The results shown are representative of the three independent experiments. *p*-value < 0.05 was considered statistically significant; individual *p*-values (** *p* < 0.01) are indicated in figures.
